# Autoantibody to apolipoprotein A-1 in hepatitis C virus infection: a role in atherosclerosis?

**DOI:** 10.1007/s12072-018-9842-5

**Published:** 2018-02-08

**Authors:** Simon H. Bridge, Sabrina Pagano, Meleri Jones, Graham R. Foster, Dermot Neely, Nicolas Vuilleumier, Margaret F. Bassendine

**Affiliations:** 1 Faculty of Health and Life Sciences, Northumbria University, Newcastle upon Tyne, UK; 2 Institute of Cellular Medicine, Newcastle University, Newcastle upon Tyne, UK; 3 Division of Laboratory Medicine, Department of Genetics and Laboratory Medicine, Geneva University Hospitals, Geneva, Switzerland; 4 The Liver Unit, Blizard Institute, Queen Mary University of London, London, UK; 5 Department of Clinical Biochemistry, Newcastle upon Tyne Hospitals NHS Foundation Trust, Newcastle upon Tyne, UK; 6 Department of Hepatology and Gastroenterology, Imperial College London, 10th Floor QEQM Wing, St. Mary’s Hospital Campus, South Wharf Street, London, W2 1NY UK

**Keywords:** Hepatitis C virus, Autoimmunity, Atherosclerosis, Cardiovascular risk, Apolipoprotein A-1

## Abstract

**Background/purpose:**

One to three per cent of the world’s population has hepatitis C virus (HCV) infection, which is not only a major cause of liver disease and cancer but also associated with an increased risk of atherosclerosis, despite an ostensibly favourable lipid profile. Autoantibodies are frequent in HCV infection and emerging evidence shows that autoantibodies could be valuable for cardiovascular disease (CVD) risk stratification. This study investigated a novel independent biomarker of CVD, autoantibodies to apolipoprotein A-1 (anti-apoA-1 IgG) and lipids in patients with chronic HCV before, during and after direct-acting anti-viral (DAA) therapy.

**Methods:**

Eighty-nine blinded serum samples from 27 patients with advanced chronic HCV were assayed for lipids and anti-apoA-1 IgG by ELISA.

**Results:**

Pre-treatment HCV viral load correlated with high-density lipoprotein cholesterol (HDL-C, *r* = 0.417; *p* = 0.042) and negatively with apolipoprotein (apo)B (*r* = − 0.497; *p* = 0.013) and markers of CVD risk, the apoB/apoA-1 ratio (*r* = − 0.490; *p* = 0.015) and triglyceride level (TG)/HDL-C ratio (*r* = − 0.450; *p* = 0.031). Fourteen (52%) of 27 patients had detectable anti-apoA-1 IgG autoantibodies pre-treatment; only two became undetectable with virological cure. Autoantibody-positive sera had lower apoA-1 (*p* = 0.012), HDL-C (*p* = 0.009) and total cholesterol (*p* = 0.006) levels.

**Conclusions:**

This is the first report of the presence of an emerging biomarker for atherosclerosis, anti-apoA-1 IgG, in some patients with HCV infection. It may be induced by apoA-1 on the surface of HCV lipoviral particles. The autoantibodies inversely correlate with apoA-1 and HDL levels and may render HDL dysfunctional. Whether these hypothesis-generating findings have clinical implications in HCV patients requires further study.

## Introduction

It is estimated that 1–3% of the world’s population has chronic hepatitis C virus (HCV) infection, which is a major cause of liver disease and liver cancer. HCV-infected patients also have increased rates of atherosclerosis, which leads to a higher risk of cardiovascular [1] and cerebrovascular morbidity and mortality [2]. The Framingham Risk Score (FRS) may underestimate coronary heart disease (CHD) risk in both HCV mono-infected and HIV/HCV co-infected persons [3] as cholesterol levels are lower in patients with chronic hepatitis C [4]. Autoantibodies are frequent in HCV infection and emerging evidence shows that autoantibodies could be of valuable help for cardiovascular risk stratification [5]. Autoantibodies against apolipoprotein A-1 (anti-apoA-1 IgG) have emerged as an independent biomarker for cardiovascular disease and mortality in several populations [6, 7]. These autoantibodies may be mediators of plaque vulnerability [8]. In rheumatoid arthritis anti-apoA-1 IgG improves the Framingham 10-year cardiovascular (CV) risk score [9]. The presence of anti-apoA-1 autoantibodies is also a risk biomarker for cardiovascular disease in type 2 diabetes mellitus [10], which is increased in HCV infection [11].

Apolipoprotein A-1 (apoA-1), the major structural protein of high-density lipoprotein (HDL), interacts with Scavenger receptor class B type 1 (SR-B1), which is the endogenous receptor for HDLs in the liver. SR-B1 is known to be involved in HCV entry and HDLs facilitate viral entry [12]. In vitro small molecule scavenger receptor BI antagonists are potent HCV entry inhibitors [13]. Another in vitro study showed that specific siRNA-mediated downregulation of apoA-I led to a reduction in both HCV RNA and viral particle levels indicating hepatitis C virus production requires apoA-1 [14]. ApoA-1 has been shown to be incorporated into HCV particles and exposed on the surface of virions [15], leading to the suggestion that HCV has developed an advantageous strategy to hijack the physiological interaction of HDL with SR-BI. This ultrastructural study confirmed that HCV particles incorporate apoB and apoE and showed that host apolipoproteins were more readily accessible to antibody labelling than HCV envelope glycoproteins. Infectious hepatitis C virions associated with these host lipoproteins are termed lipoviral particles (LVP). The lipoprotein components of LVP not only play key roles in viral attachment and entry, but also reduce the sensitivity of HCV to neutralising antibodies and mask viral epitopes [16, 17].

This study investigated serum lipids and the novel independent biomarker of CVD, autoantibodies to apolipoprotein A-1 (anti-apoA-1 IgG) in patients with chronic HCV before, during and after direct-acting anti-viral (DAA) therapy.

## Materials and methods

### Ethics

Ethical approval was obtained from City Road and Hampstead (formerly Moorfields and Whittington) Research Ethics Committee for serial blood sampling of patients with chronic hepatitis C virus infection undergoing antiviral therapy.

### Patients

Eighty-nine non-fasting serum samples from 27 patients with advanced chronic HCV [*n* = 15 genotype (GT) 1, 2 with mixed cryoglobulinemia (MC) and *n* = 12 GT3, 2 with MC (Table 1)] who had received treatment with sofosbuvir-based direct-acting antiviral (DAA) therapy were assayed for anti-apoA-1 IgG and serum lipids. In HCV GT1 patients sofosbuvir was combined with ledipasvir (+ ribavirin in 10/15 and + pegylated IFN in 1/15) and in HCV GT3 patients it was combined with daclatasvir in 11/12 and with ribavirin and pegylated IFN in 1/12.

**Table 1:** Serum lipids in 27 patients with advanced chronic hepatitis C infection before and at the end (week 12) of direct-acting antiviral therapy

Characteristics	HCV genotype 1, *n* = 15			HCV genotype 3, *n* = 12		
	Baseline	Week 12	*p* value*	Baseline	Week 12	*p* value*
Sex, *n* (M/F)		11/4	–		7/5	–
Age (yr), median (range)	62	(48–74)	–	56	(36–65)	–
HCV RNA log10 (IU/ml), mean ± SD	5.80 ± 0.82	0	–	5.33 ± 0.74	0	–
Total cholesterol (mmol/l), median (IQR)	4.25 (1.75)	4.30 (1.15)	*0.662*†	4.10 (1.18)	4.40 (1.90)	*0.652*†
LDL-C (mmol/l), mean ± SD	2.27 ± 0.83	2.10 (0.74)	*0.666*‡	2.37 ± 0.84	2.48 ± 0.76	*0.762*†
Apolipoprotein B (g/l), median (IQR)	0.79 (0.49)	0.76 (0.24)	*0.512*†	0.87 (0.47)	0.86 (0.42)	*0.722*†
HDL-C (mmol/l), mean ± SD	1.44 ± 0.48	1.56 ± 0.46	*0.511*‡	1.26 ± 0.40	1.36 ± 0.44	*0.603*‡
Apoprotein A1 (g/l), mean ± SD	1.63 ± 0.29	1.63 ± 0.43	*0.972*‡	1.38 ± 0.30	1.50 ± 0.39	*0.432*‡
Triglyceride (mmol/l), median (IQR)	1.20 (1.40)	1.10 (0.90)	*0.456*†	1.25 (0.55)	1.50 (0.90)	*0.773*†
ApoB/apoA1 ratio, median (IQR)	0.46 (0.39)	0.46 (0.35)	*0.593*†	0.62 (0.24)	0.51 (0.25)	*0.657*†
TG/HDL-C ratio, median (IQR)	0.92 (1.01)	0.69 (0.89)	*0.505*†	1.08 (0.99)	1.00 (0.82)	*0.653*†
TC/HDL-C ratio, median (IQR)	2.71 (2.70)	2.63 (1.92)	*0.437*†	3.23 (1.66)	3.39 (1.63)	*0.838*†
Anti-apoA1 antibodies^a^, median (IQR)	34.20 (27.70)	37.30 (27.23)	*0.565*†	45.60 (32.40)	37.00 (32.43)	*0.510*†

ApoB/apoA1, apolipoprotein B/apolipoprotein A1 ratio

Parametric variables shown as the mean plus/minus standard deviation. Non-parametric variables shown as the median and the interquartile range

*IQR* interquartile range, *SD* standard deviation; *TC/HDL-C* total cholesterol/high-density lipoprotein cholesterol, *TG/HDL-C* triglyceride/high-density lipoprotein cholesterol

*p values presented are for the comparison between variables at baseline and week 12. *p* < 0.05 was considered significant

†*p* value calculated using the non-parametric Kruskal-Wallis test

‡*p* value calculated using the parametric *t* test

^a^The anti-ApoA-1 antibody positivity cutoff was predefined at 37%, which corresponded to an OD_405 nm_ of 0.6 as previously validated and described (18–20)

**Fig. 1:**
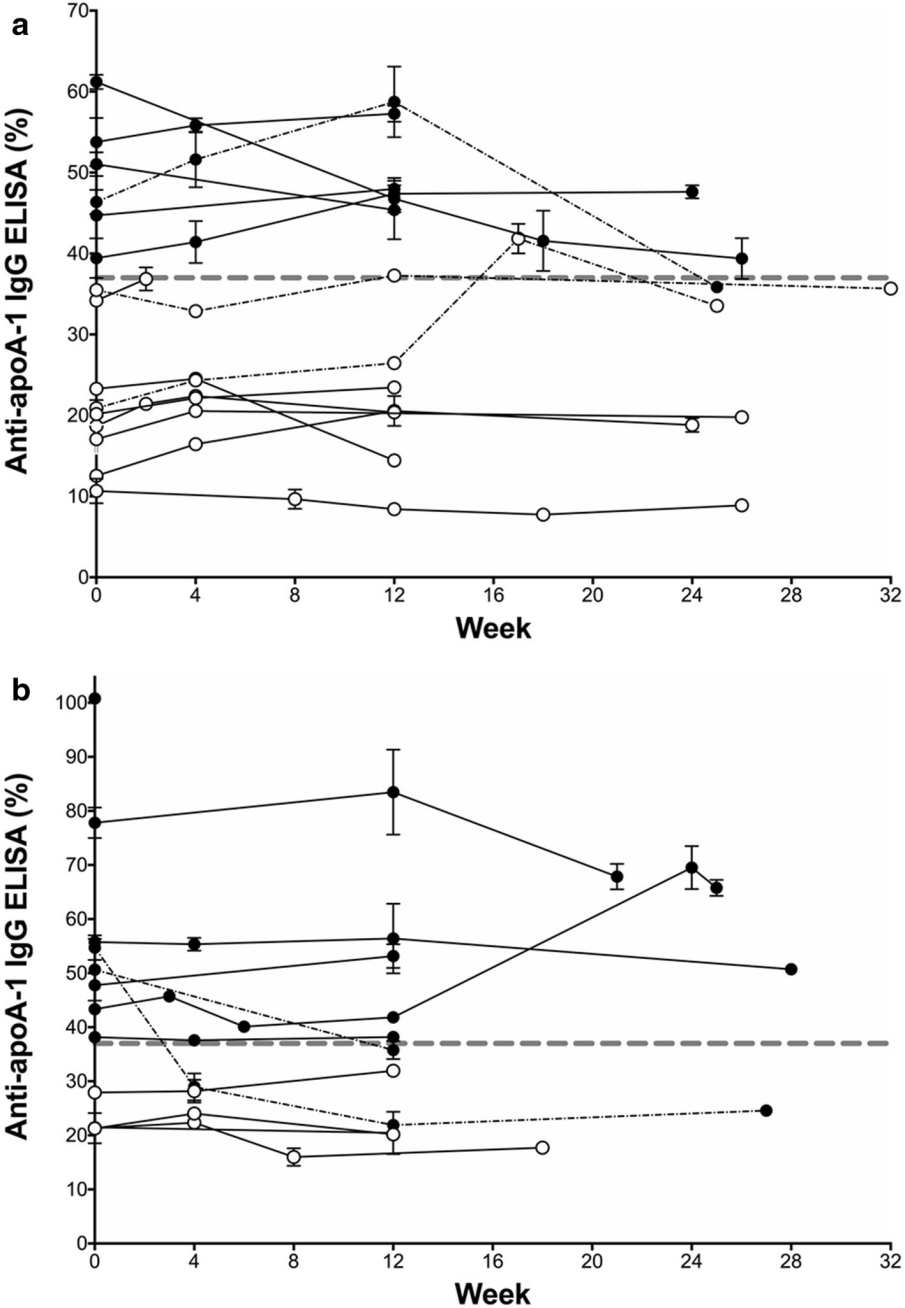
Autoantibody to apolipoprotein A-1 IgG ELISA reading (cutoff 37%) in 27 patients with advanced chronic HCV before, during and after direct-acting anti-viral therapy, commenced at week 0. By week 12 all patients responded to antiviral treatment and were HCV RNA negative. **a** Results in HCV genotype 1 patients, 2/15 relapsed after end of therapy; **b** results in HCV genotype 3 patients, 1/12 relapsed. Closed black circles show autoantibody-positive patients at week 0, open circles show autoantibody negative patients at week 0, the broken dashed black line shows patients that changed from seropositive to seronegative and vice versa during sampling. Error bars show the standard deviation between replicate values. The thick dashed grey line indicates a positive AAA1 IgG response

Twenty of 27 of the patients had established cirrhosis and 2 of these had type 2 diabetes mellitus. Twenty-four of 27 were non-responders to previous course(s) of interferon (IFN)-based anti-viral therapy. The samples were obtained before, during and after treatment; 76/89 serum samples were from 20/27 patients (between 3 and 6 samples per patient) and 12/89 serum samples were from 6/27 patients (2 samples per patient); the remaining 1/89 serum sample was obtained pre-treatment but there was insufficient serum available at later time points for analysis (see Fig. 1 for serial samples in each patient). All 89 samples were labelled randomly and assayed ‘blind’ to patient source and the results of DAA therapy.

### Lipid profiles

Total cholesterol, triglyceride (TG) and HDL-cholesterol (HDL-C) levels were measured by automated enzymatic methods and low-density lipoprotein (LDL) cholesterol was estimated indirectly with the Friedewald equation [(LDL-cholesterol) = (total cholesterol) – (HDL-cholesterol) – (TG)/2.2]. Apolipoproteins A-I and B were measured by automated immunoturbidometric methods on a Roche Cobas Modular c702 analyser (Roche Diagnostics, Lewes, UK).

### Anti-apoA-1 IgG ELISA

Serum samples were assayed for anti-apoA-1 antibody levels as previously described [6, 8, 18, 19]. Briefly, Maxi-Sorb plates (Nunc) were coated with purified, human-derived delipidated apoA-1 (20 µg/ml; 50 μl/well) for 1 h at 37 °C. After three washes with phosphate-buffered saline (PBS)/2% bovine serum albumin (BSA; 100 μl/well), all wells were blocked for 1 h with 2% BSA at 37 °C. Samples were diluted 1:50 in PBS/2% BSA and incubated for 60 min. Additional patient samples at the same dilution were also added to an uncoated well to assess individual nonspecific binding. After six further washes, 50 μl/well of signal antibody (alkaline phosphatase-conjugated anti-human IgG; Sigma-Aldrich) diluted 1:1000 in PBS/2% BSA solution was incubated for 1 h at 37 °C. After six more washes (150 μl/well) with PBS/2% BSA solution, the phosphatase substrate p-nitrophenyl phosphate disodium (50 μl/well; Sigma-Aldrich) dissolved in diethanolamine buffer (pH 9.8) was added. Each sample was tested in duplicate and absorbance, determined as the optical density at 405 nm (OD_405_ nm), was determined after 20 min of incubation at 37 °C (VersaMax, Molecular Devices). The corresponding nonspecific binding value was subtracted from the mean absorbance value for each sample. The positivity cutoff was predefined as previously validated and set at an OD value of 0.6 and 37% of the positive control value as described earlier [18–20]. At the cutoff level, the intra- and inter-assay coefficients of variation were shown to be 16% (*n* = 10) and 12% (*n* = 8), respectively [20].

### Statistical analyses

Statistical analysis was performed with the use of Minitab 17 (Minitab Ltd, Coventry, UK) and GraphPad Prism 7 (GraphPad, La Jolla, USA). The distribution of continuous data variables was assessed by normality tests. Normally distributed continuous variables were reported as means ± standard deviations and compared between groups using two-sample *t* tests. Non-parametrically distributed variables were reported as median and interquartile ranges and compared between groups using Kruskal-Wallis tests. The correlation between two continuous variables was assessed using either Pearson’s correlation coefficient test for parametric data or Spearman’s rank correlation test for non-parametric ranked data variables. The strength of the association (*R*^2^) between groups was assessed with regression analysis. For all analyses *p* < 0.05 was considered significant.

## Results

Blinded serial serum samples (*n* = 89) from 27 patients with chronic hepatitis C infection undergoing DAA therapy were assayed for viral load, lipids and anti-apoA-1 IgG (AAA1). The clinical characteristics of the patients at baseline and week 12 are summarised in Table 1. There was no significant difference in the serum lipid concentrations at week 0 and 12 of DAA therapy, when all 27 patients had a virological response (undetectable HCV RNA).

Pre-treatment (week 0) HCV viral load positively correlated with high-density lipoprotein cholesterol (HDL-C, *r* = 0.417; *p* = 0.042) and negatively with apoB (*r* = − 0.497; *p* = 0.013) and LDL-C (*r* = − 0.494; *p* = 0.017). Ratios associated with increased cardiovascular disease risk were also negatively correlated with the HCV RNA:apoB/apoA-1 ratio (*r* = − 0.490; *p* = 0.015), TG/HDL-C ratio (*r* = − 0.450; *p* = 0.031) and total cholesterol/HDL-C ratio (*r* = − 0.450; *p* = 0.027).

**Fig. 2:**
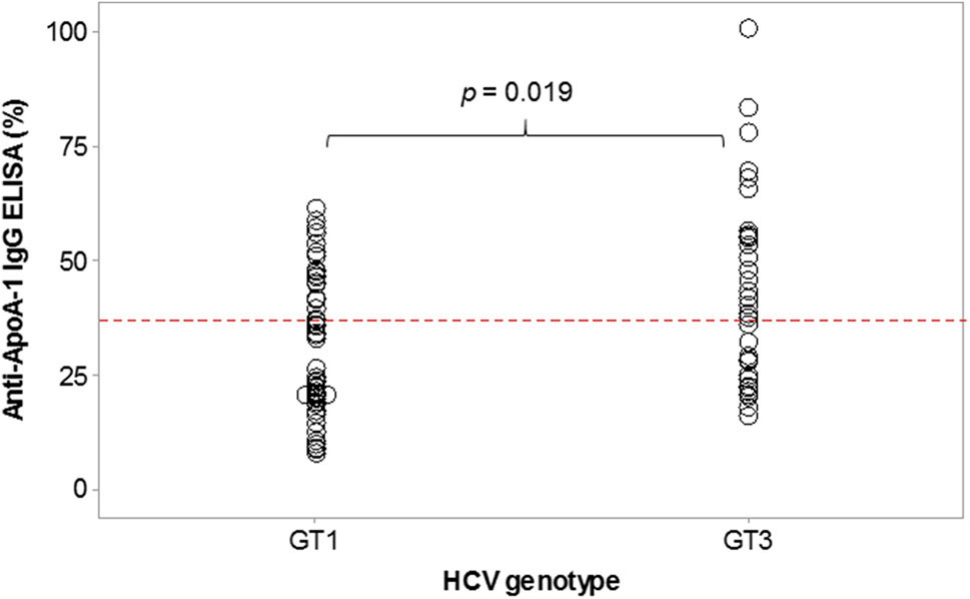
Level of serum autoantibody to apolipoprotein A-1 IgG by ELISA [cutoff 37% and shown by the broken red line] in advanced chronic HCV according to HCV genotype. HCV GT1 (*n* = 52 samples, 31 HCV RNA negative and 21 with detectable HCV RNA). HCV GT3 (*n* = 37 samples, 22 HCV RNA negative and 15 with detectable HCV RNA). Higher AAA1 antibody levels were found in HCV GT3 patients compared to HCV GT1 patients (38.2 vs. 33.2%; *p* = 0.019)

Fourteen of the 27 (52%) patients with chronic HCV (6 GT1, 8 GT3) were found to be seropositive for AAA1 antibodies at baseline prior to receiving DAA therapy. Twelve patients were positive for AAA1 antibodies at the end of DAA therapy at week 12. Longitudinal AAA1 analysis before, during and after response to DAA treatment showed that overall 41 (46%) out of 89 serum samples were positive for AAA1 IgG antibodies. Ten (71%) of the 14 patients were consistently positive for ApoA-1 IgG, five (83%) out of the six patients with HCV genotype 1 infection (Fig. 1a) and five (63%) out of the eight patients with HCV genotype 3 infection (Fig. 1b). A further two HCV GT3 patients who were positive for AAA1 antibodies by ELISA became seronegative following virological cure. Two GT1 patients were intermittently AAA1 seropositive and then returned to seronegative. Furthermore, when comparing the AAA1 antibody levels in the 89 serum samples and stratifying according to the two HCV genotypes there was a significant difference in the overall levels of serum AAA1 autoantibodies between GT1 and GT3 patients (Fig. 2, *p* = 0.019).

Anti-apoA-1 IgG was found in two of four patients with mixed cryoglobulinemia (MC). To confirm the specificity of binding, the apoA-1 immunoreactivity was further analysed in four random HCV serum samples; it was shown to be specifically inhibited with peptide F3L1 but not with a scrambled peptide as previously described [20].

**Fig. 3:**
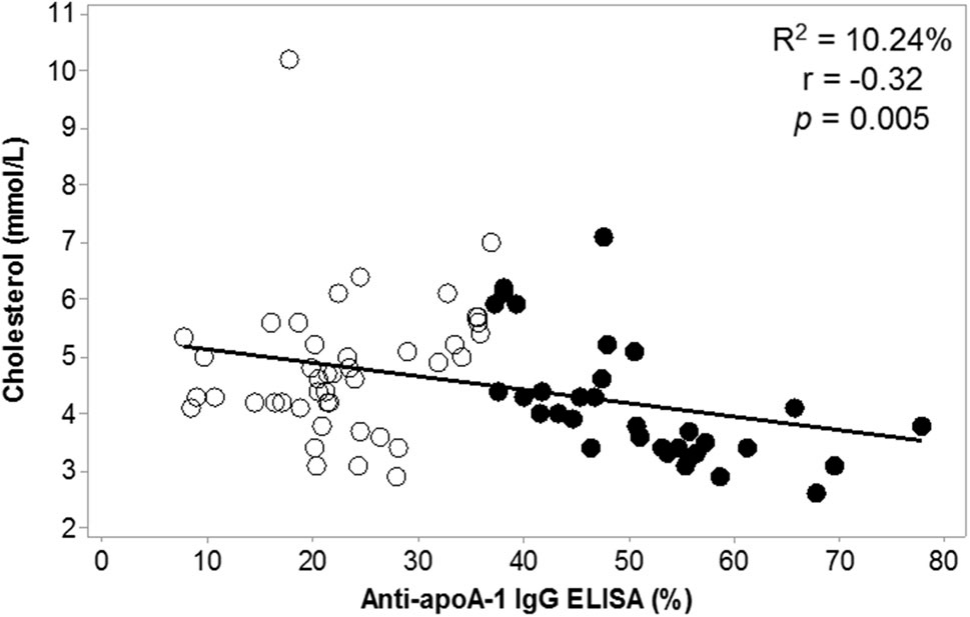
IgG autoantibody response to apoA-1 is associated with cholesterol concentration. Correlation analysis showed an inverse relationship between AAA1 autoantibody responses and cholesterol concentration (*r* = − 0.32; *p* = 0.005). Linear regression analysis showed that the magnitude of the AAA1 autoantibody response was a significant negative predictor of cholesterol concentration (*R*^2^ = 10.24; *p* = 0.005). Open circled samples were AAA1 IgG-negative samples and closed black circles were AAA1 IgG-positive samples

Of the 89 serial serum samples evaluated for AAA1 antibodies and lipids, 41 were identified as AAA1 IgG positive and the remaining 48 were seronegative for AAA1 antibodies. A comparison of the lipoprotein characteristics of the seropositive and -negative samples is summarised in Table 2. Eighteen of the 41 anti-apoA-1 IgG-positive sera had detectable HCV RNA (5.04 ± 1.3 log_10_ IU/ml). In the 41 AAA1-positive sera, Spearman correlation analysis using ranked variables showed that the magnitude of the autoantibody response inversely correlated with cholesterol (*p* = 0.005), HDL-C (*p* = 0.025), apoA-1 (*p* = 0.014) and apoB (*p* = 0.040). Linear regression analyses showed that the magnitude of the AAA1 autoantibody response was a significant negative predictor of cholesterol concentration (Fig. 3, *R*^2^ = 10.24%; *p* = 0.005).

**Table 2:** Comparison of characteristics between anti-apolipoprotein-A1 (AAA1) IgG-positive samples and AAA1 negative samples

Characteristic	Anti-apolipoprotein Al seropositive, *n* = 41	Anti-apolipoprotein Al seronegative, *n* = 48	*p* value *
HCV RNA log_10_ (IU/ml), mean ± SD	2.27 ± 2.67	1.93 ± 2.68	0.552†
Total cholesterol (mmol/l), median	3.90 (1.10)	4.70 (1.18)	**0.006**
(IQR) LDL-C (mmol/l), median (IQR)	2.16 (1.55)	2.32 (1.27)	0.308‡
Apolipoprotein B (g/l), median (IQR)	0.79 (0.48)	0.85 (0.41)	0.063‡
HDL-C (mmol/l), mean ± SD	1.25 ± 0.39	1.54 ± 0.64	**0.009**
Apolipoprotein A1 (g/l), mean ± SD	1.40 ± 0.33	1.60 ± 0.42	**0.012**
Triglyceride (mmol/l), median (IQR)	1.30 (0.65)	1.20 (0.93)	0.341‡
ApoB/apoA1 ratio, median (IQR)	0.52 (0.37)	0.50 (0.35)	0.557‡
TG/HDL-C ratio, median (IQR)	1.00 (1.00)	0.85 (1.00)	0.105‡
TC/HDL-C ratio, median (IQR)	3.33 (2.17)	3.11 (2.96)	0.873‡
Anti-apoA1 antibodies^a^, median (IQR)	48.00 (14.30)	21.50 (8.84)	**<** **0.001**

ApoB/apoA1, apolipoprotein B/apolipoprotein A1 ratio; HDL-C, high-density lipoprotein cholesterol

Parametric variables shown as the mean ± standard deviation. Non-parametric variables shown as the median and interquartile range

*LDL-C* low-density lipoprotein cholesterol, *IQR* interquartile range, *OD* optical density, *SD* standard deviation, *TC/HDL-C* total cholesterol/high-density lipoprotein cholesterol, *TG/HDL-C* triglyceride/high-density lipoprotein cholesterol

*p values presented are for the variable comparison between AAA1 seropositive samples and AAA1 seronegative samples. *p* < 0.05 was considered significant

†*p* value calculated using the parametric *t* test

‡*p* value calculated using the non-parametric Kruskal-Wallis test

^a^The anti-ApoA-1 antibody positivity cutoff was predefined at 37%, which corresponded to an OD_405 nm_ of 0.6 as previously validated and described (18–20)

**Fig. 4:**
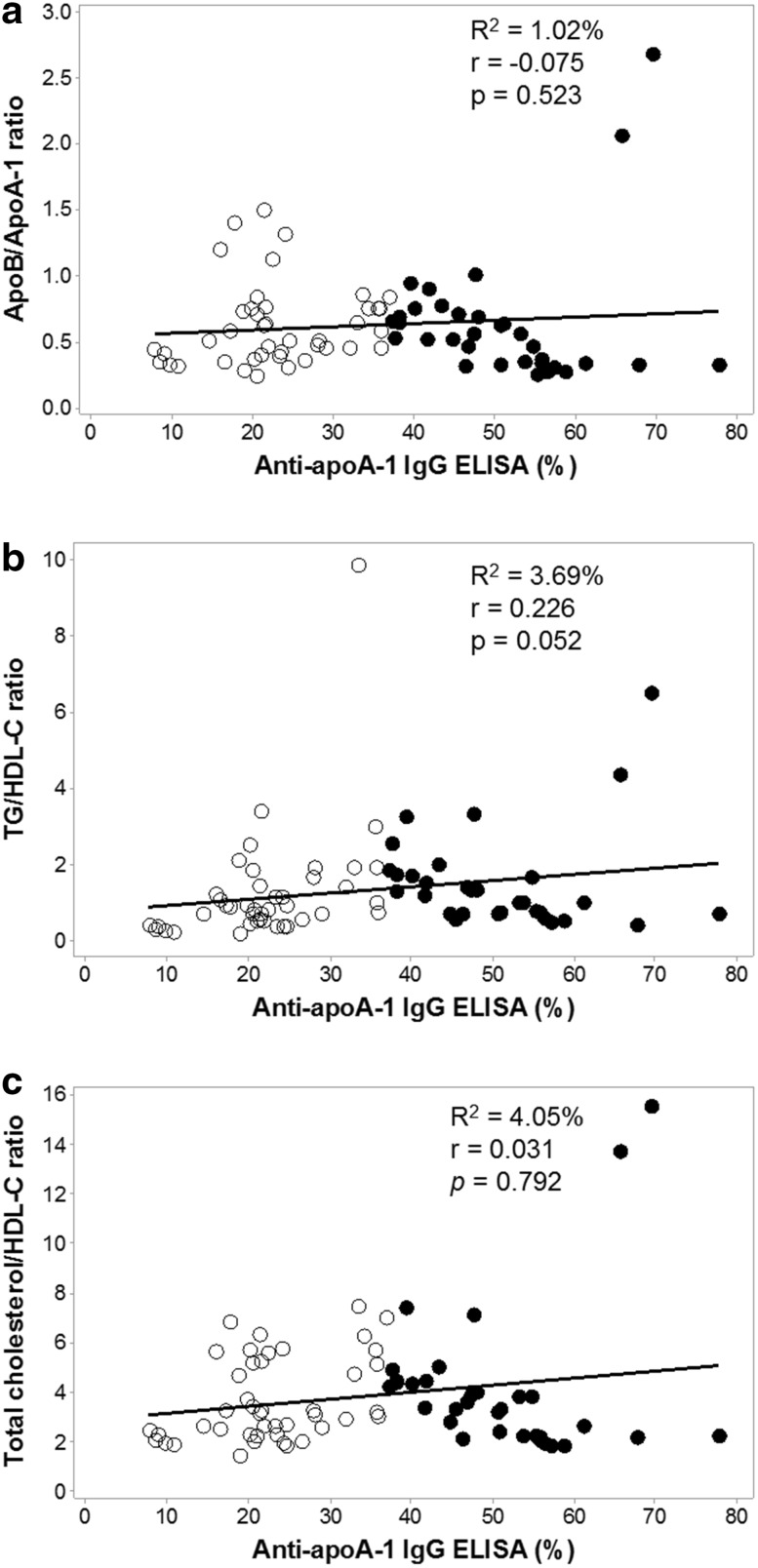
IgG autoantibody response to apoA-1 is not associated with traditional lipid cardiovascular risk factors; **a** shows no significant relationship with the proatherogenic:antiatherogenic ratio apoB/apoA-1, **b** shows no significant relationship with TG/HDL-C and **c** shows no significant relationship with the total cholesterol/HDL-C ratio. Open circled samples were AAA1 IgG-negative samples and closed black circles were AAA1 IgG-positive samples

As anti-apoA-1 is a novel biomarker of CVD, we determined whether there is any correlation of traditional lipid ratios associated with increased CVD risk with the presence of anti-apoA-1 IgG. At pre-treatment there was no correlation of anti-apoA-1 IgG with the apoB/apoA-1 ratio (*r* = − 0.236; *p* = 0.268), TG/HDL-C ratio (*r* = 0.148; *p* = 0.499) and total cholesterol/HDL-C ratio (*r* = − 0.151; *p* = 0.481). Overall, the magnitude of the AAA1 response showed no correlation with these lipid CVD risk ratios, as shown in Fig. 4.

## Discussion

Epidemiological studies have shown HCV is associated with an increased risk of cardiovascular and cerebrovascular mortality as well as peripheral arterial disease (reviewed in [2]). HCV is associated with an ostensibly favourable lipid profile with an accompanying low classical risk score for atherosclerosis, so it is unclear how best to assess cardiovascular risk [21]. Anti-ApoA-1 IgG is an emerging prognostic cardiovascular marker [22] and in this ‘proof of concept’ study we have shown for the first time that around half of patients with chronic HCV have these autoantibodies.

Previous studies using the same ELISA to detect serum anti-apoA-1 IgG have found it in a proportion of healthy subjects (0–6.5%) without CVD or autoimmune disease (reviewed in [22] [23]). In a large population-based study (*n* = 6649 subjects) the prevalence of anti-apoA-1 IgG in the general population was 19.9 % and was significantly associated with CVD [odds ratio 1.34, *p* = 0.018], independently of established CV risk factors [24]. In this large population based study anti-apoA-1 IgG levels independently predicted all-cause mortality and were found to be linked to *FCRL3*, a susceptibility gene for numerous autoimmune diseases [25], which encodes a member of the immunoglobulin receptor superfamily.

The presence of anti-apoA-1 IgG in the serum of around half of these patients with chronic HCV is similar or higher than has been reported in other diseases [20]. For example, in patients with periodontitis, where it is a biomarker of atherosclerosis burden, the prevalence of anti-apoA-1 IgG was found to be 23.8% compared to 6.5% in age- and sex-matched controls (*p* = 0.009) [23]. In patients with rheumatoid arthritis anti-apoA-1 IgG positivity was 17% and was associated with a higher incidence of major cardiovascular events [9]. In a recent study in type 2 diabetes the incidence of autoantibodies to apoA-1 was 37.5% in patients with cardiovascular disease (CVD) and patients who were autoantibody positive showed 5.7 times increased CVD risk [10].

Anti-apoA-I IgG is thus recognised as an independent predictor of poor cardiovascular outcome in several different populations at risk for CVD with and without concomitant autoimmune disease and provides incremental prognostic information over traditional risk factors for CVD [20]. It would be of considerable interest if this novel biomarker is predictive of atherosclerosis and atherosclerotic plaque vulnerability in the setting of chronic HCV where assessment of CV risk remains an unmet clinical need.

In this study we found higher serum levels of anti-apoA-1 IgG in patients with HCV genotype 3 infection, which needs confirmation in a larger cohort. HCV GT3 is associated with a higher risk of early death and liver-related clinical events [26] and we have previously reported genotype-specific differences in regulation of lipoproteins [27]. Anti-ApoA-1 IgG serum levels have been found to predict worse post-stroke outcomes [28] and HCV is associated with increased cerebrovascular morbidity and mortality [2].

Generally, HDL and its principal protein component, apoA-1, are considered to be atheroprotective. We confirmed that AAA1 antibody-positive sera had significantly lower serum levels of both HDL and apoA-1, implying a possible role in atherogenesis through HDL dysfunction. We also confirmed pre-treatment that the hepatitis C viral load correlated negatively with LDL, the ‘bad’ cholesterol, and with other ‘classic’ CVD risk ratios. For example, the ApoB/apoA-1 ratio has been proposed as a simple, accurate risk factor for cardiovascular disease [29]—the lower the apoB/apoA-I ratio, the lower is the risk. However, we found the lower the ratio, the higher the viral load pre-treatment, so this simple ‘marker’ may underestimate CVD risk in HCV infection. Overall, there was no significant association of traditional lipid CVD risk factors with anti-apoA-1 IgG in this cohort, which reflects not only the profound effect of HCV on lipid parameters but also the effect of this autoantibody on HDL-C and cholesterol levels.

As expected [4], these patients had low/normal serum cholesterol levels and we found no significant rise at the end of DAA treatment (EOT, week 12). However, patients with chronic HCV whose sera were anti-apoA-1 IgG positive had significantly lower total serum cholesterol levels, suggesting that these autoantibodies are modulating lipid metabolism in chronic hepatitis C. The concept of HCV hijacking the machinery responsible for controlling lipid metabolism to improve viral fitness has recently been supported by a study of the effect of direct-acting anti-viral therapy on the peripheral and hepatic lipid metabolism [30]. Rapid changes in the serum lipoprotein particle concentration during DAA treatment were observed including an increase in LDL early in therapy, reflecting a shift in lipid metabolism in the setting of inhibition of HCV replication and implicating a direct viral effect on serum lipoprotein concentrations. This study also found that rapid changes in the apoB/apoA-1 ratio during DAA therapy were not sustained at EOT [30], similar to our findings at week 12 of DAA therapy.

In contrast to these dynamic changes in serum lipoprotein composition and concentration during DAA therapy, we found that the presence or absence of autoantibody to apoA-1 remained largely stable during sofosbuvir-based antiviral therapy and after virological cure over a short follow-up period. This is similar to experience in children with chronic hepatitis C receiving IFN-based anti-viral therapy where autoantibodies were found to be common at baseline, during and after treatment [31]. The vast majority of our patient cohort had received prior IFN-based anti-viral therapy and IFN may induce autoimmune disorders or worsen pre-existing autoimmune disorders [32], so it will be important to establish whether the prevalence of anti-apoA-1 IgG is similar in untreated HCV infection. Sofosbuvir plus daclatasvir has recently been shown to be effective in patients with HCV-associated cryoglobulinemia vasculitis with a fall in cryoglobulin levels after antiviral therapy but an increased number of T-regulatory cells [33]. It is thus of interest that we found only 2/14 patients with positive anti-apoA-1 IgG pre-treatment became seronegative with virological cure. It is possible that the continuing presence of autoantibodies after cure [31] contributes to the increase in regulatory T cells, which have a role in the suppression of autoimmune responses.

Previous work has shown that the anti-apoA-I autoantibody response is strongly biased towards the C-terminal alpha-helix of the protein [20], which plays an important role in lipid binding. These autoantibodies may thus modulate binding of hepatitis C LVP to SR-B1 as it has been shown that various lipoprotein receptors redundantly participate in HCV entry, in a manner dependent on the lipoproteins associated with HCV particles [34]. ApoA-1 is exposed on the surface of infectious HCV lipoviral particles [15] and these autoantibodies may be exerting anti-viral activity, similar to downregulation of apoA-1 [14].

The link between HCV infection and atherosclerosis is intriguing and pathogenic mechanisms remain elusive as HCV has such a profound effect on lipid components of the Framingham Risk Score and traditional lipid biomarkers of CVD. While our results should be interpreted with caution given the small sample size of the study, we suggest that apoA-1 on the surface of HCV lipoviral particles may lead to the development of autoantibodies to this host lipoprotein in predisposed individuals. It will be important to establish whether these autoantibodies develop during acute HCV infection where the titre of IgG antibody to HCV proteins only rises by the 4th to 6th month after infection [35]. The high prevalence of these autoantibodies in chronic HCV infection may be linked to the increased risk of atherosclerosis and associated morbidity and mortality.
